# Association between the triglyceride–glucose index and left atrial appendage thrombogenic milieu in patients with atrial fibrillation

**DOI:** 10.3389/fcvm.2025.1472183

**Published:** 2025-09-29

**Authors:** Hanze Sun, Shuangzheng Hu, Rongrong Pan, Qin Zhuang, Yi Zheng

**Affiliations:** ^1^ Department of Cardiology, Cixi People Hospital Medical Health Group (Cixi People Hospital), Ningbo, China; ^2^ Department of Ultrasonography, Cixi People Hospital Medical Health Group (Cixi People Hospital), Ningbo, China

**Keywords:** atrial fibrillation, triglyceride-glucose index, insulin resistance, left atrial appendagethrombus, spontaneous echo contrast

## Abstract

**Objectives:**

The triglyceride–glucose (TyG) index is an independent predictor of atrial fibrillation (AF). The presence of left atrial appendage (LAA) thrombogenic milieu (LAATM) is associated with ischemic stroke in patients with AF. The present study aimed to explore the relationship between the TyG index and LAATM in patients with AF.

**Methods:**

Patients with nonvalvular AF who were admitted to the Department of Cardiology at Cixi People Hospital Medical Health Group for catheter ablation or LAA closure from April 2018 to January 2024 were retrospectively included. The study population was divided into two groups on the basis of the presence (LAATM group) or absence (non-LAATM group) of LAATM as determined by preprocedural transesophageal echocardiography. The relationship between the TyG index and LAATM was observed.

**Results:**

A total of 466 patients with nonvalvular AF who underwent transesophageal echocardiography (TEE) examination were included. LAATM was observed in 62 (13.3%) patients. The TyG index was higher in patients with LAATM (7.56 ± 0.59 vs. 7.04 ± 0.47, *P* < 0.001). Multivariate logistic analysis demonstrated that higher TyG index was an independent predictor of LAATM development (odds ratio = 12.78, 95% confidence interval 2.95–55.42; *P* < 0.001). Receiver operating characteristic curve analysis revealed that the optimal cutoff value of the TyG index for predicting LAATM development was 7.01 (area under the curve: 0.76; sensitivity 93.5%, specificity 50.2%).

**Conclusion:**

The TyG index, which is calculated on the basis of triglyceride and fasting plasma glucose levels, was positively associated with LAATM development in patients with AF.

## Introduction

Atrial fibrillation (AF) is the most prevalent type of arrhythmia in the clinic (1, 2). Patients with AF have a greater risk of ischemic stroke (3), and the majority of thrombi form in the left atrial appendage (LAA) (4). Previous investigations have demonstrated that the presence of LAA thrombogenic milieu (LAATM), which includes thrombi, sludge and spontaneous echo contrast (SEC), is significantly associated with stroke in patients with AF (5–7). Some factors that are related to the development of LAATM, including comorbidities, echocardiographic parameters and laboratory biomarkers, have been reported (8–10).

Insulin resistance (IR) has been proven to be associated with AF development and recurrence after catheter ablation (11–14). IR can cause endothelial dysfunction by decreasing nitric oxide production by endothelial cells and increasing the release of procoagulant factors that cause platelet aggregation (15). Therefore, IR may increase the risk of thrombus formation in patients with AF. However, data on the association of IR with LAATM are limited. The triglyceride-glucose (TyG) index, which is assessed on the basis of triglyceride (TG) and fasting plasma glucose (FPG) levels, is now widely used in the assessment of IR (16). In this study, we evaluated the association between the TyG index and LAATM in patients with AF.

## Methods

### Study population

Patients with nonvalvular AF who were admitted to the Department of Cardiology at Cixi People Hospital Medical Health Group for catheter ablation or LAA closure from April 2018 to January 2024 were retrospectively included. All patients with transesophageal echocardiography (TEE) examination were screened. Patients with missing data for TyG index calculation were excluded. Patients with mechanical valves or moderate-to-severe mitral stenosis, or uncontrolled bleeding diseases, or creatinine clearance (CrCl) < 30 ml/min were also excluded. Demographic data and comorbid conditions, including congestive heart failure, hypertension, diabetes, previous stroke or transient ischemic attack (TIA) and previous bleeding, were collected to evaluate the risk of stroke (CHA_2_DS_2_-VASc score) and bleeding (HAS-BLED score). Fasting blood samples were collected within 24 h of admission. FPG and lipid indices, including total cholesterol (TC), TG, high-density lipoprotein cholesterol (HDL-C) and low-density lipoprotein cholesterol (LDL-C) levels, were measured and recorded. The TyG index was calculated as follows: TyG index = ln [TG (mg/dl) × FPG (mg/dl)/2]. This study was conducted in compliance with the law protecting personal data and in accordance with the guidelines of the Declaration of Helsinki, and it was approved by the Ethics Committee of Cixi People Hospital Medical Health Group (2024-LP-LW001).

### Echocardiographic examination

Every patient was treated with direct oral anticoagulants (DOACs) after admission to the hospital. Low-dose DOACs were used in some special subset of patients, such as patients aged ≥ 75 years, weighted < 50 kg, or with CrCl < 50 ml/min (2). Transthoracic echocardiography and TEE were performed within 48 h before the procedure. The left atrial (LA) diameter and left ventricular ejection fraction (LVEF) were measured. Signs of LAATM, including LAA thrombi, sludge, and dense SEC, were detected by transesophageal echocardiography. LAA thrombus was defined as an echodense mass with uniform tissue that was different than that of the LA endocardial wall. SEC was defined as an echogenic, swirling pattern of blood flow observed under the standard gain setting during the cardiac cycle, and it was graded according to the Fatkin classification. SEC grade of 3 or 4 was considered dense. LAA sludge was defined as a dynamic, viscid, layered echo dense finding without a discrete mass. LAA sludge is considered to represent a stage between SEC and thrombus formation (17).

### Statistical analysis

The study population was divided into two groups based on the absence (non-LAATM group) or presence (LAATM group) of LAATM. Normally distributed continuous variables are expressed as the mean ± standard deviation (SD) and were compared via the *t* test, whereas nonnormally distributed variables are expressed as the median [interquartile range (IQR)] and were compared via the Mann–Whitney U test. Categorical variables are expressed as percentages and were compared via the chi-square test or Fisher's exact test where appropriate.

Logistic regression analyses were performed to explore the potential risk factors for LAATM development. A univariate model without adjusted factors was initially performed. The variables with a *P* value < 0.05 in the univariate model were included in the multivariate logistic regression analysis. Odds ratios (ORs) and 95% confidence intervals (CIs) were calculated. A receiver operating characteristic (ROC) curve was used to assess the ability of the TyG index to predict the development of LAATM. The optimal cutoff value and the area under the curve (AUC) were determined on the basis of Youden's index. All the statistical analyses were performed with SPSS 22.0 (IBM, Armonk, NY, USA), and a *P* value < 0.05 (2-tailed) was considered statistically significant.

## Results

### Baseline characteristics

**Figure 1:**
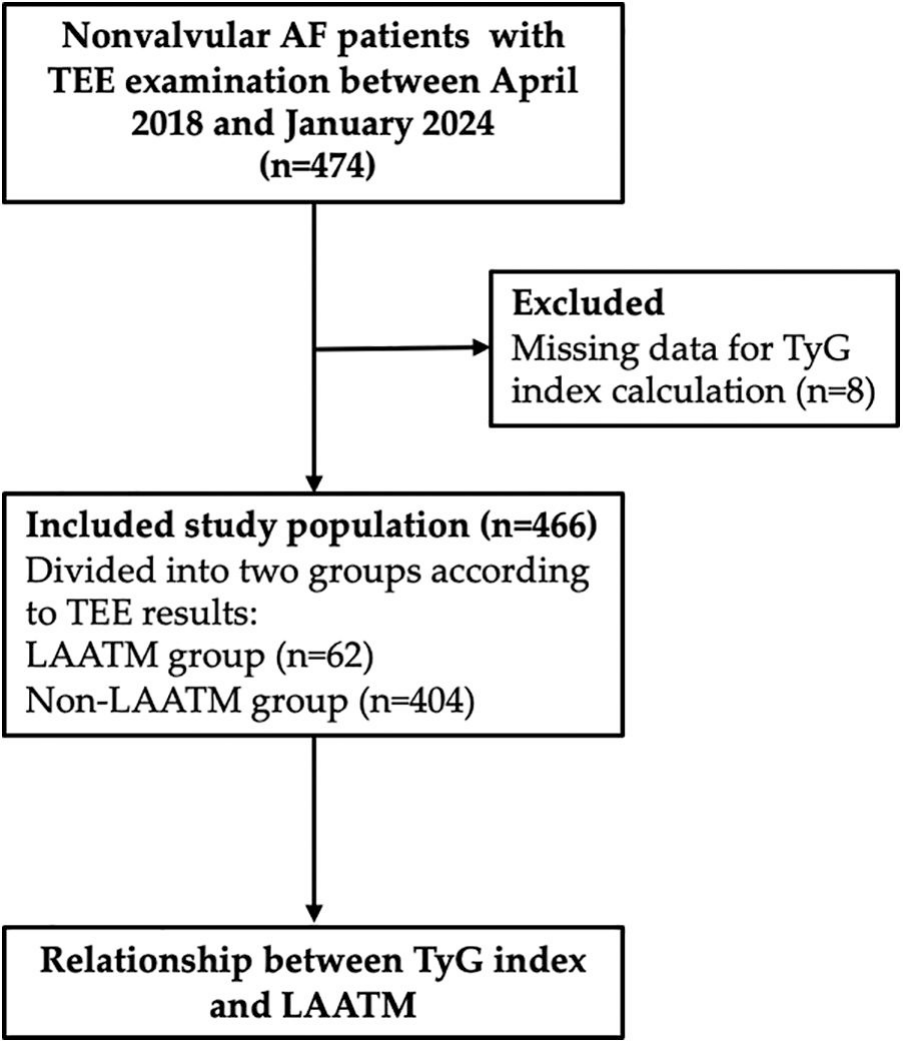
Study flow chart. A total of 474 nonvalvular AF patients with TEE examination were screened between April 2018 and January 2024. Eight patients were excluded due to missing data for TyG index calculation. Finally, a total of 466 patients were included and further divided into LAATM group (*n* = 62) and non-LAATM group (*n* = 404) according to the TEE results. AF, atrial fibrillation; TEE, transesophageal echocardiography; TyG index, triglyceride–glucose index; LAATM, left atrial appendage thrombogenic milieu.

A total of 466 patients with nonvalvular AF who underwent TEE examination were included for analysis after 8 patients excluded due to missing data for TyG index calculation (Figure 1). LAATM was observed in 62 (13.3%) patients (LAATM group). The baseline characteristics of the participants are shown in Table 1. The rate of patients treated with Low-dose DOACs was comparable between LAATM group and non-LAATM group (12.9% vs. 14.9%, *P* = 0.686). The percentage of patients with paroxysmal AF was greater in the non-LAATM group (32.9% vs. 12.9%, *P* = 0.005). The comorbid conditions were similar between the groups. The CHA_2_DS_2_-VASc score and HAS-BLED score were comparable between the non-LAATM group and the LAATM group. The LA diameter was greater in the LAATM group (48.0 ± 6.6 mm vs. 44.5 ± 7.4 mm, *P* < 0.001). The FPG (5.49 ± 0.63 mmol/L vs. 5.15 ± 1.00 mmol/L, *P* < 0.001) and TG [1.87 (1.44–2.99) mmol/L vs. 1.35 (1.00–1.74), *P* < 0.001] levels were greater in patients with LAATM.

**Table 1:** Baseline characteristics of the study population.

Variables	Non-LAATM group *n* = 404	LAATM group *n* = 62	*P* value
Age, mean ± SD, years	69.7 ± 8.7	68.2 ± 9.9	0.212
Male sex, *n* (%)	261 (64.6)	40 (64.5)	0.989
Paroxysmal AF, *n* (%)	133 (32.9)	8 (12.9)	0.005
Body mass index, mean ± SD, kg/m^2^	24.2 ± 3.5	24.2 ± 3.7	0.969
Low-dose direct oral anticoagulants, *n* (%)	60 (14.9)	8 (12.9)	0.686
Comorbid conditions			
Congestive heart failure, *n* (%)	54 (13.4)	9 (14.5)	0.805
Hypertension, *n* (%)	256 (63.4)	44 (71.0)	0.245
Diabetes, *n* (%)	37 (9.2)	5 (8.1)	0.779
Coronary artery disease, *n* (%)	35 (8.7)	7 (11.3)	0.501
Previous stroke/TIA, *n* (%)	42 (10.4)	8 (12.9)	0.553
Previous bleeding, *n* (%)	21 (5.2)	3 (4.8)	1.000
Clinical scoring point			
CHA_2_DS_2_-VASc score, median (IQR), points	2 (1–3)	2 (1–3)	0.456
HAS-BLED score, median (IQR), points	1 (1–1)	1 (1–1)	0.598
Echocardiographic data			
LA diameter, mean ± SD, mm	44.5 ± 7.4	48.0 ± 6.6	<0.001
LVEF, mean ± SD, %	62.0 ± 6.6	60.9 ± 6.2	0.219
Laboratory data			
FPG, mean ± SD, mmol/L	5.15 ± 1.00	5.49 ± 0.63	<0.001
TC, mean ± SD, mmol/L	3.69 ± 1.05	4.01 ± 1.26	0.065
TG median (IQR), mmol/L	1.35 (1.00–1.74)	1.87 (1.44–2.99)	<0.001
HDL-C, mean ± SD, mmol/L	1.15 ± 0.34	1.06 ± 0.21	0.067
LDL-C, mean ± SD, mmol/L	2.15 ± 0.76	2.36 ± 0.80	0.044
TyG index, mean ± SD	7.04 ± 0.47	7.56 ± 0.59	<0.001

AF, atrial fibrillation; FPG, fasting plasma glucose; HDL-C, high-density lipoprotein cholesterol; IQR, interquartile range; LA, left atrial; LAATM, left atrial appendage thrombogenic milieu; LVEF, left ventricular ejection fraction; LDL-C, low-density lipoprotein cholesterol; SD, standard deviation; TC, total cholesterol; TG, triglyceride; TIA, transient ischemic attack; TyG index, triglyceride‒glucose index.

### The TyG index and LAATM

**Figure 2:**
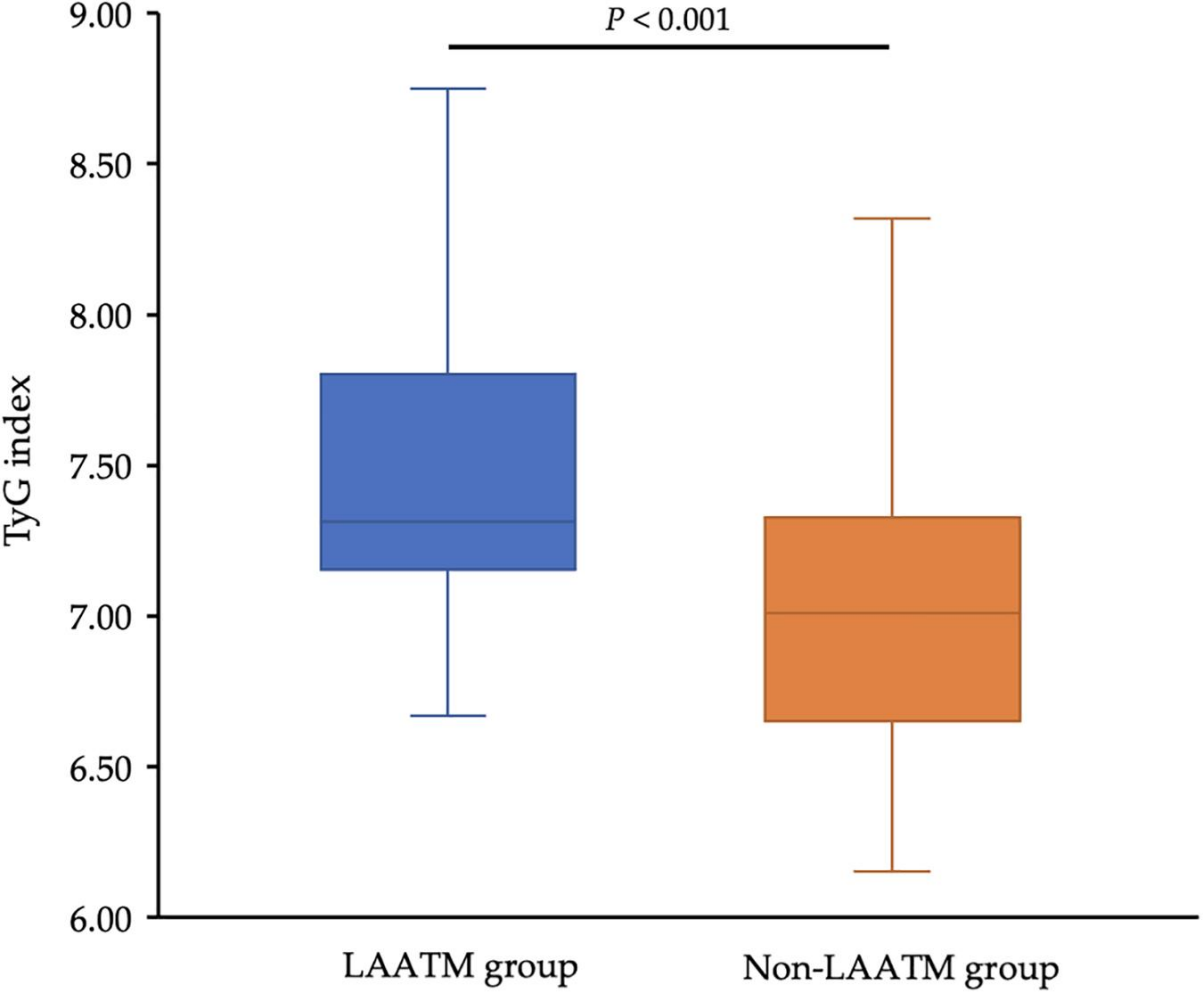
Comparison of the TyG index between the LAATM group and the non-LAATM group. The TyG index was higher in the LAATM group (7.56 ± 0.59; blue bar) compared with non-LAATM group (7.04 ± 0.47; orange bar) (*P* < 0.001). TyG index, triglyceride–glucose index; LAATM, left atrial appendage thrombogenic milieu.

The TyG index was higher in patients with LAATM (7.56 ± 0.59 vs. 7.04 ± 0.47, *P* < 0.001; Table 1 and Figure 2). Univariate logistic regression analysis revealed that paroxysmal AF and increased HDL-C levels were negatively associated with LAATM, whereas an increased LA diameter, higher FPG, TC, TG, and LDL-C levels, and an increased TyG index were positively associated with LAATM development in patients with nonvalvular AF. After multivariate analysis, a higher TyG index was an independent predictor of LAATM development (OR = 12.78, 95% CI 2.95–55.42; *P* < 0.001). In addition, patients with paroxysmal AF were still at lower risk compared to those with persistent AF (OR = 0.40, 95% CI 0.17–0.94; *P* = 0.035) (Table 2).

**Table 2:** Logistic regression analysis of risk factors for LAATM.

Variables	Univariate model		Multivariate model	
	OR (95% CI)	*P* value	OR (95% CI)	*P* value
Age	0.98 (0.95–1.01)	0.213	–	–
Male sex	1.00 (0.57–1.74)	0.989	–	–
Paroxysmal AF	**0.30** (**0.14–0.65)**	**0**.**002**	**0.40 (0.17–0.94)**	**0.035**
Body mass index	1.00 (0.93–1.08)	0.969	–	–
Congestive heart failure	1.10 (0.51–2.36)	0.805	–	–
Hypertension	1.41 (0.79–2.54)	0.246	–	–
Diabetes	0.87 (0.33–2.31)	0.870	–	–
Coronary artery disease	1.34 (0.57–3.17)	0.503	–	–
Previous stroke/TIA history	1.28 (0.57–2.87)	0.553	–	–
Previous bleeding history	0.93 (0.27–3.21)	0.905	–	–
CHA_2_DS_2_-VASc score	0.88 (0.70–1.11)	0.287	–	–
HAS-BLED score	1.11 (0.75–1.63)	0.616	–	–
LA diameter	**1.06** (**1.03–1.10)**	**0**.**001**	1.04 (0.996–1.09)	0.074
LVEF	0.98 (0.94–1.02)	0.236	–	–
FPG	**1.35** (**1.06–1.71)**	**0**.**014**	0.81 (0.54–1.20)	0.285
TC	**1.29** (**1.02–1.64)**	**0**.**033**	0.98 (0.46–2.08)	0.949
TG	**2.19** (**1.67–2.88)**	**<0**.**001**	0.79 (0.47–1.33)	0.383
HDL-C	**0.30** (**0.10–0.95)**	**0**.**041**	0.78 (0.32–1.93)	0.593
LDL-C	**1.41** (**1.01–1.99)**	**0**.**046**	0.78 (0.27–2.28)	0.655
TyG index	**6.51** (**3.72–11.38)**	**<0**.**001**	**12.78 (2.95–55.42)**	**0.001**

AF, atrial fibrillation; CI, confidence interval; FPG, fasting plasma glucose; HDL-C, high-density lipoprotein cholesterol; LA, left atrial; LAATM, left atrial appendage thrombogenic milieu; LVEF, left ventricular ejection fraction; LDL-C, low-density lipoprotein cholesterol; OR, odds ratio; TC, total cholesterol; TG, triglyceride; TIA, transient ischemic attack; TyG index, triglyceride‒glucose index.

The bold values indicated statistical significance.

**Figure 3:**
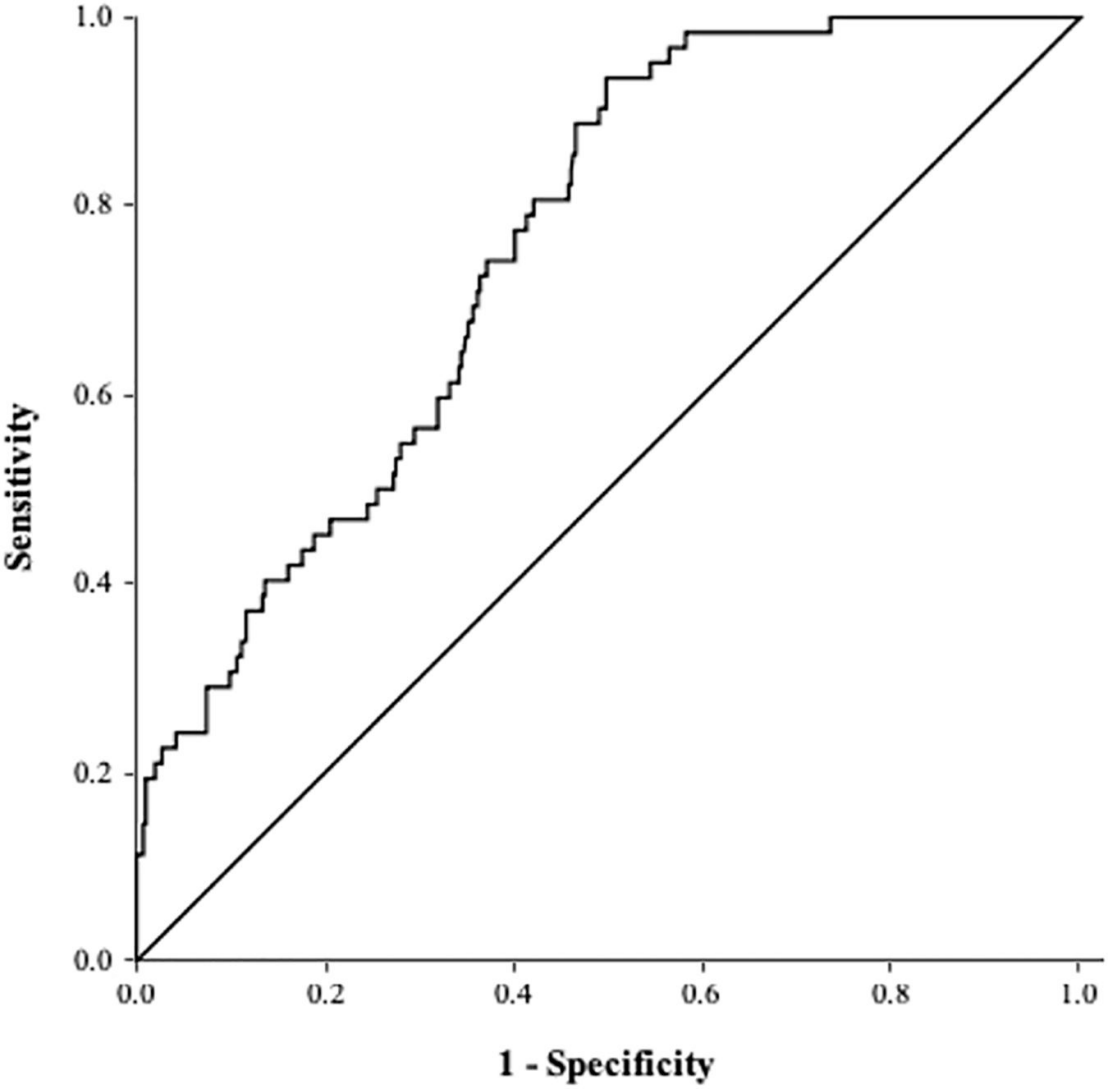
ROC curve of the TyG index for predicting LAATM. The area under the curve was 0.76 (95% CI 0.70–0.81, *P* < 0.001). The optional cutoff value of the TyG index was 7.01 (sensitivity 93.5%, specificity 50.2%). TyG index, triglyceride–glucose index; LAATM, left atrial appendage thrombogenic milieu.

ROC curve analysis revealed an AUC of 0.76 (95% CI 0.70–0.81, *P* < 0.001; Figure 3). The optimal cutoff value of the TyG index for predicting LAATM development was 7.01 (sensitivity 93.5%, specificity 50.2%).

## Discussion

To our knowledge, the present study is the first to report the relationship between IR and LAATM development in patients with AF. The results revealed that the TyG index was significantly greater in patients with AF and LAATM. Multivariate logistic regression analysis demonstrated that a higher TyG index was an independent predictor of LAATM development.

In patients with IR, more insulin production is needed to maintain normal blood glucose levels. The hyperinsulinemic-euglycemic clamp (HEC) method is considered the best method, but this technique is rather complicated, less practical, and quite expensive. The TyG index, which is calculated according to two low-cost and common laboratory parameters (TG and FPG levels), is now widely used for assessing IR. Therefore, the TyG index has been considered a surrogate marker of IR. A previous investigation demonstrated that the TyG index was strongly associated with HEC (AUC = 0.858), with a TyG index cutoff of 4.68 having a sensitivity of 96.5% and a specificity of 85% for estimating IR in the adult population (16). Data have shown that a higher TyG index is associated with a greater risk of cardiovascular diseases, including coronary artery disease (18–20) and heart failure (21–23). In most studies, the TyG index was calculated based on TG and FPG levels from a single point-in-time measurement at baseline, regardless of their changes over time, which may lead to potential bias. Recently, Cui et al. showed that the risk of cardiovascular diseases development increased along with the quartile of the cumulative TyG index (24). They also found that the cumulative effect of the TyG index seemed to be independent and better than the TyG index at baseline in predicting cardiovascular diseases. Therefore, evaluating the mean changes in the TyG index in cardiovascular diseases progression and follow-up is warranted in future studies.

AF is the most sustained type of arrhythmia in the clinical setting (1). The TyG index has been reported to be related to AF (11, 12). A retrospective observational study including 356 hospitalized patients revealed that an increased TyG index is an independent risk factor for AF (11). A meta-analysis demonstrated that the TyG index was higher in patients with AF than in their non-AF counterparts (12). Catheter ablation is an effective method for treating AF and maintaining sinus rhythm. Several studies have shown that a higher TyG index is associated with a higher recurrence rate after ablation (14, 25). Wang et al. (24) reported that the TyG index was independently associated with AF recurrence following *de novo* ablation. Similarly, Tang et al. (14) demonstrated that an elevated preablation TyG index was associated with an increased risk of late AF recurrence after ablation in non-diabetic patients.

AF significantly increases the risk of ischemic stroke (3). The most commonly used scoring system (CHA_2_DS_2_-VASc score) for assessing stroke risk in patients with AF includes diabetes (1). Previous studies have shown that an elevated TyG index is associated with ischemic stroke (26, 27). However, no data on the relationship between the TyG index and ischemic stroke in patients with AF have been reported thus far. The majority of thrombi associated with ischemic stroke originate in the LAA (4). SEC in the LAA is also strongly associated with stroke in patients with AF (5). Some comorbidities (heart failure, chronic kidney disease, previous stroke or TIA), echocardiographic parameters (LA diameter, E/e’, or LAA velocity) and laboratory biomarkers (B-type natriuretic peptide, C-reactive protein, or D-dimer) were shown to be predictors of LAATM development (8–10). Some studies have also shown that patients with diabetes have a greater risk of LAATM development (28, 29). However, no investigation has been performed to explore the association of IR with LAATM. In the present study, we found that a higher TyG index, which is a surrogate marker of IR, was an independent predictor of LAATM development. The ROC analysis indicated that the TyG index exhibited a high sensitivity (93.5%) but low specificity (50.2%), which may result in a substantial number of false positives. LAATM is significantly associated with stroke in patients with AF. As a predictor for LAATM, it should at least have a high sensitivity to screen the patients with potential high risk of LAATM to undergo TEE. Once LAATM was detected, oral anticoagulants could be prescribed to the patients on time to reduce the risk of stroke in the clinical practice.

Some potential mechanisms may explain the relationship between IR and LAATM. First, insulin fails to increase cyclic adenosine monophosphate levels within platelets in patients with IR, thus impairing its antiaggregation effects (30). IR also reduces the sensitivity of platelets to the antiaggregation effects of nitric oxide and prostaglandin I2, which in turn alters calcium influx and promotes platelet aggregation (31). Second, previous studies have reported dysfunctions in intrinsic coagulation that are associated with hyperglycemia and hyperinsulinemia (32, 33). Increased synthesis of coagulant factors (FXII, FXI and FIX) in hepatocytes along with a shorter activated partial thromboplastin time were observed in patients with impaired insulin sensitivity, and these effects are probably mediated by a low-grade inflammatory reaction that is induced by IR (34). Third, inflammation may play a role in the relationship between IR and LAATM. A cross-sectional study revealed an association between the systemic immune-inflammation index and IR (35). Previous studies have demonstrated that there is an apparent link between inflammation and thrombogenesis (36, 37).

There were several limitations of our study. First, this was a single-center, retrospective, observational study which may carry an inherent risk of selection bias. The exact causal relationship between the TyG index and LAATM development is unknown. In addition, the LAATM group comprised a small number of cases. Therefore, the results should be proved by further multicenter prospective studies with large study population. Second, the TyG index was derived from a single point-in-time measurement of TG and FPG levels after hospitalization, which does not account for intra-individual variation or reflect long-term metabolic stability; this may lead to possible bias due to measurement error. Due to the retrospective design, blood sampling and TEE examination were not performed at the same day for some patients. To our knowledge, most previous studies regarding the relationship between laboratory parameters and LAATM also had this limitation. The time interval in the present study was within 24 h in most patients. Therefore, we believe that time interval between blood sampling and TEE examination should not or only slightly affect the relationship between TyG index and LAATM. Third, the study population was limited to AF patients who were candidates for catheter ablation or LAA closure. Therefore, our results cannot be extended to all AF patients. Further investigations should be conducted to prove the findings of the present study in broader AF population.

## Conclusion

The TyG index, which is an easily calculated, cost-effective, and valid surrogate marker of IR, was an independent risk factor for LAATM development in patients with AF.

## Data Availability

The original contributions presented in the study are included in the article/Supplementary Material, further inquiries can be directed to the corresponding author.
